# Superparamagnetic Iron Oxide Nanoparticles Modified with Silica Layers as Potential Agents for Lung Cancer Treatment

**DOI:** 10.3390/nano10061076

**Published:** 2020-05-31

**Authors:** Katarzyna Reczyńska, Marta Marszałek, Arkadiusz Zarzycki, Witold Reczyński, Kamil Kornaus, Elżbieta Pamuła, Wojciech Chrzanowski

**Affiliations:** 1 Faculty of Materials Science and Ceramics, AGH University of Science and Technology, Al. Mickiewicza 30, 30-059 Kraków, Poland; kmr@agh.edu.pl (K.R.); wreczyn@agh.edu.pl (W.R.); kornaus@agh.edu.pl (K.K.); 2 Sydney Nano Institute, Faculty of Medicine and Health, Sydney School of Pharmacy, The University of Sydney, Pharmacy and Bank Building, Camperdown, NSW 2006, Australia; 3 Institute of Nuclear Physics, Polish Academy of Sciences, ul. Radzikowskiego 152, 31-342 Kraków, Poland; marta.marszalek@ifj.edu.pl (M.M.); arkadiusz.zarzycki@ifj.edu.pl (A.Z.)

**Keywords:** lung cancer, nanoparticles, nanoformualtions, controlled drug release, drug delivery, superparamagnetic iron oxide nanoparticles (SPIONs), silica coating, iron release, nano-bio-characterization, Atomic Force Microscopy

## Abstract

Superparamagnetic iron oxide nanoparticles (SPIONs) are promising drug delivery carriers and hyperthermia agents for the treatment of cancer. However, to ensure their safety in vivo, SPIONs must be modified in order to prevent unwanted iron release. Thus, SPIONs were coated with silica layers of different morphologies: non-porous (@SiO_2_), mesoporous (@mSiO_2_) or with a combination of non-porous and mesoporous layers (@SiO_2_@mSiO_2_) deposited via a sol–gel method. The presence of SiO_2_ drastically changed the surface properties of the nanoparticles. The zeta potential changed from 19.6 ± 0.8 mV for SPIONs to −26.1 ± 0.1 mV for SPION@mSiO_2_. The Brunauer–Emmett–Teller (BET) surface area increased from 7.54 ± 0.02 m^2^/g for SPIONs to 101.3 ± 2.8 m^2^/g for SPION@mSiO_2_. All types of coatings significantly decreased iron release (at least 10 fold as compared to unmodified SPIONs). SPIONs and SPION@mSiO_2_ were tested in vitro in contact with human lung epithelial cells (A549 and BEAS-2B). Both nanoparticle types were cytocompatible, although some delay in proliferation was observed for BEAS-2B cells as compared to A549 cells, which was correlated with increased cell velocity and nanoparticles uptake.

## 1. Introduction

Superparamagnetic iron oxide nanoparticles (SPIONs) consist of magnetite (Fe_3_O_4_) or maghemite (γ-Fe_2_O_3_). The methods of SPIONs synthesis are well established, enabling the fabrication of uniform nanoparticles of the desired size and shape [1,2]. SPIONs have already been explored for a vast variety of biomedical purposes, including magnetic resonance imaging (MRI) [3,4], tissue engineering [5,6] and cell separation [7]. Due to their unique magnetic properties, they gained a lot of interest in the field of controlled drug delivery [8,9], photodynamic therapy [10,11] and hyperthermia [12,13], thus SPIONs are regarded as one of the most promising candidates for the development of novel methods for cancer treatment. Numerous studies utilized SPIONs for magnetically mediated hyperthermia—a concept based on the fact that the viability of malignant cells is lower at temperatures above 41 °C in comparison to non-malignant cells [14,15]. SPIONs can be accumulated directly at the tumor site and heated up using an external alternating magnetic field [13]. The unique magnetic properties of SPIONs, e.g., high magnetization values and superparamagnetism, make them particularly useful as hyperthermia agents [16]. The efficacy of such a treatment may be increased by the simultaneous delivery of anticancer drugs or biologically active molecules. Lachowicz et al. [17] synthesized SPIONs decorated with curcumin conjugated with sodium alginate. The nanoparticles with an average diameter of 50 nm were proved to be suitable for hyperthermia treatment. Mansouri et al. [18] encapsulated paclitaxel in nanoparticles composed of palmitoyl chitosan and SPIONs. The study showed that the release profile of paclitaxel was changed by the application of a magnetic field. The developed hybrid nanoparticles enhanced the apoptosis of MCF-7 cells due to hyperthermia effects coupled with an increased concentration in paclitaxel in cells.

Conventionally, SPIONs are destined for subcutaneous or intravenous administration [19]. However, in the case of lung cancer, pulmonary delivery of the drug is more effective, allows for the reduction of systemic toxicity of the treatment and increases patients’ compliance [20]. Sadhukha et al. [21] showed that it is possible to effectively deliver epidermal growth factor (EGFR) targeted SPIONs directly to the lungs via inhalation, resulting in a uniform distribution of the nanoparticles in the lungs. The administration of EGFR-targeted SPIONs was followed by magnetic hyperthermia, which not only significantly inhibited tumor growth, but also did not cause any damage to healthy tissues or other side effects. Another advantage of using SPIONs as pulmonary drug delivery carriers is the possibility for the guided accumulation of the nanoparticles only in the affected parts of the lungs using an external magnetic field. Price et al. [22] fabricated SPION-loaded lactose microparticles containing doxorubicin and confirmed the feasibility of the targeted accumulation of SPIONs in selected lung lobes in mice. They found that the amount of SPIONs in a magnetized left lung lobe was 10 times higher than in the right lobe.

Despite those promising results, some issues connected with the use of SPIONs for the treatment of lung cancer still need to be solved. One of the major concerns is related to the dissolution of SPIONs and the possible release of iron that can actually promote cancer growth [23]. If SPIONs are supposed to be delivered directly to the tumor site and be used for the induction of hyperthermia and the killing of the cancer cells, they should not supply those cells with iron and stimulate their growth at the same time. Therefore, it is indispensable to modify their surface to prevent iron release. Unfortunately, the majority of the studies focused on SPIONs neglect that aspect. Surface modifications of SPIONs are mostly aimed at the improvement of their colloidal stability (coating with poly(ethylene glycol)—PEG [24] or poly(vinyl alcohol)—PVA [25]), biocompatibility (coating with natural polymers such as alginate [26] or chitosan [18]) or the attachment of various biologically active ligands (functionalization with PEG or gold [27]). However, in terms of the inhibition of iron release, the modification of SPIONs with stable silica coatings seems to be the most appropriate. Such coatings are commonly used to protect materials from corrosion [28] and offer numerous advantages, including the ease of deposition and microstructure modification [29]. Furthermore, SPIONs were already extensively tested in vitro in contact with different cell types (e.g., MCF-7 breast cancer cell line [30], primary human umbilical cord vein endothelial cells (HUVECs) [31] or in different types of malignant cell lines containing folic acid receptors (FARs) [32]). However, the information on the influence of SPIONs on lung epithelial cells of malignant or non-malignant origin is yet scarce.

Hence, the aim of this study was to modify SPIONs in order to develop a versatile platform for lung cancer diagnosis and therapy. SPIONs were coated with silica layers with different microstructures that were supposed to: (1) decrease iron release from the nanoparticles, (2) generate a high surface area available for further loading with anticancer drugs or other biologically active moieties, and (3) maintain their magnetic properties suitable for biomedical applications. Modified SPIONs were evaluated using various physicochemical methods. In the end, their cytocompatibility with lung epithelial cells was also determined.

## 2. Materials and Methods

### 2.1. Materials

Superparamagnetic iron (II,III) oxide nanoparticles (SPIONs), tetraethyl orthosilicate (TEOS) and hexadecyltrimethylammonium bromide (CTAB) were purchased from Sigma-Aldrich (St. Louis, MO, USA). Ethanol (99.9%), ammonia (28%), potassium ferrocyanide and hydrochloric acid (35%) were of analytical grade and were purchased from Avantor Performance Materials, Poland S.A. Ultra-high quality water (UHQ-water) was produced in a UHQ-PS purification system (Elga, High Wycombe, UK). All reagents were used without additional purification.

The liquid mixture used for the fabrication of the silica coating consisted of ethanol (99.8%), UHQ-water and ammonia (28%) (further referred to as Et/UQH/Am) in the following volumetric ratio: ethanol/UHQ-water/ammonia 32:8:1.

### 2.2. Modification of SPIONs

**Figure 1:**
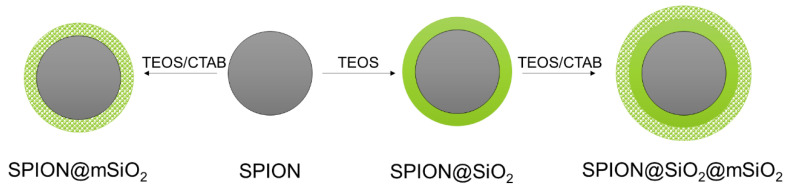
Schematic representation of the modification of superparamagnetic iron oxide nanoparticles (SPIONs).

SPIONs were coated with different types of silica layers: non-porous (@SiO_2_), mesoporous (@mSiO_2_) or with a combination of non-porous and mesoporous layers (@SiO_2_@mSiO_2_) (Figure 1). Silica layers were deposited on the SPIONs with the use of TEOS as a silica precursor and CTAB as a pore former (for @mSiO_2_ coatings only) using a sol–gel technique [28,33].

#### 2.2.1. Non-Porous Silica Coating (@SiO_2_)

One gram of SPIONs was suspended in 200 mL of Et/UHQ/Am and homogenously dispersed using an ultrasound probe (40% amplitude, 30 min, Vibra-Cell, Sonic & Materials, Inc., Newtown, CT, USA). One milliliter of TEOS was added dropwise into the suspension and ultrasound homogenization was continued for 5 min. The suspension was then aliquoted into polypropylene falcon tubes and vigorously mixed using a vortex mixer (500 rpm, VX-200, Labnet International, Big Flats, NY, USA) for 12 h. The obtained silica coated SPIONs (SPION@SiO_2_) were collected by solid–liquid separation using an external neodymium magnet, washed three times with 25 mL ethanol and three times with 25 mL UHQ-water, followed by air drying overnight at 60 °C.

#### 2.2.2. Mesoporous Silica Coating (@mSiO_2_)

One gram of SPIONs or SPION@SiO_2_ was suspended in 175 mL Et/UHQ/Am and homogenized using an ultrasound probe (40% amplitude, 15 min). One gram of CTAB, previously dissolved in 25 mL Et/UHQ/Am, was added into the suspension. Ultrasound homogenization was continued for another 15 min followed by the dropwise addition of 1 mL of TEOS. After 5 min of ultrasound homogenization, the suspension was aliquoted into polypropylene falcon tubes and was vigorously mixed using a vortex mixer (500 rpm) for 12 h. Mesoporous silica coated SPIONs (SPION@mSiO_2_) or SPIONs coated with combined non-porous and mesoporous silica coatings (SPION@SiO_2_@mSiO_2_) were collected, washed three times with 25 mL ethanol and three times with 25 mL UHQ-water and air dried overnight at 60 °C. Additional thermal treatment of SPIONs with @mSiO_2_ coatings (dwelling at 250 °C, 2 h) was performed to remove trace remnants of CTAB.

### 2.3. Physiochemical Characterization

#### 2.3.1. Fourier Transform Infrared Spectroscopy (FTIR)

FTIR of the nanoparticles was performed using a Bruker Tensor 27 spectrophotometer (Bruker). SPIONs were embedded in KBr pellets and the transmission spectra were recorded at 4000–425 cm^−1^ with a 4 cm^−1^ resolution. For each sample, 64 single scans were collected. The obtained spectra were analyzed using OPUS software.

#### 2.3.2. Magnetic Properties

The magnetic properties of the nanoparticles were investigated using a SQUID magnetometer (Quantum Design MPMS XL, San Diego, CA, USA). Accurately weighted nanoparticles (12 mg) were placed inside a gelatin capsule and immobilized by GE Varnish glue (Cryospares™, Abingdon, UK). The magnetization of the nanoparticles was measured at 300 K as a function of a magnetic field in the range of ±50 kOe.

#### 2.3.3. Transmission Electron Microscopy (TEM)

The nanoparticles were suspended in UHQ-water and sonicated for 5 min (30% amplitude). The suspension droplets were put on formvar-coated copper grids (200 mesh, Creative Bioarray, Shirley, NY, USA). After blotting and drying, the specimens were observed with an FEI Tecnai 12 Biotwin microscope (FEI, Hillsboro, OR, USA) using a Gatan Orius SC1000 CCD camera (Gatan, Inc., Pleasanton, CA, USA).

#### 2.3.4. Atomic Force Microscopy (AFM)

The morphology and surface stiffness of the SPIONs and SPION@mSiO_2_ were evaluated using AFM. The nanoparticles were suspended in UHQ-water and dispersed using an ultrasound probe (20% amplitude, 10 min). A drop of suspension was then placed on a clean mica disc (Agar Scientific, Stansted, UK) coated with poly-L-lysine (0.1%, Sigma-Aldrich, St. Louis, MO, USA) for 5 min. Excess nanoparticles were washed from the discs using UHQ-water and the remaining nanoparticles were dried in nitrogen flow at room temperature. The topography and phase images of the nanoparticles were recorded using a NanoIR™ (Anasys Instruments, Santa Barbara, CA, USA) in tapping mode using a silicon nitride probe with a spring constant of 40 N/m (EXT125, AppNano, Mountain View, CA, USA) at a scan rate of 0.5 Hz.

#### 2.3.5. Surface Zeta Potential

The surface zeta potential of the nanoparticles was measured using a LiteSizer 500 (AntonPaar, Austria). The nanoparticles were suspended in UHQ-water (30–50 µg/mL) and homogenously dispersed using an ultrasound probe (20% amplitude, 10 min). The suspension was placed in Omega cuvettes (AntonPaar, Graz, Austria) and three measurements (50 runs for each) were recorded for each sample. The results were analyzed using dedicated Kalliope software (version 1.2.0, AntonPaar, Graz, Austria).

#### 2.3.6. Surface Area

The surface area of the nanoparticles was evaluated using the Brunauer–Emmett–Teller (BET) method. Accurately weighted nanoparticles were degassed at room temperature for 24 h to obtain 2 µmHg pressure. The surface area was determined via the multipoint nitrogen adsorption method (ASAP 2000, Micrometric, Norcross, GA, USA).

#### 2.3.7. Iron Release

Fifty milligrams of the nanoparticles were placed in sealed dialysis bags (ZelluTrans/Roth, MWCO: 10–12 kDa, Carl Roth GmbH + Co. KG, Karlsruhe, Germany) immersed in 25 mL of UHQ-water in falcon tubes. The nanoparticles were incubated for 24 h at 37 °C with constant shaking (the tubes were mounted in a horizontal shaker (50 rpm VX-200, Labnet International, Big Flats, NY, USA). The experiment was run in triplicate (three samples for each type of the nanoparticles). The concentration of iron released from the nanoparticles to the surrounding UHQ-water was determined using atomic absorption spectroscopy, electrothermal technique (ET AAS, Perkin Elmer 4100 ZL with Zeeman background correction, Waltham, MA, USA). The measurement was performed using a hollow-cathode lamp (HCL, λ: 248.3 nm, aperture: 0.2 nm), the temperature of sample decomposition was set to 1400 °C and sample atomization to 2400 °C. Each sample was measured in duplicate.

### 2.4. In Vitro Cytotoxicity

SPIONs and SPION@mSiO_2_ were selected for the evaluation of in vitro cytotoxicity in contact with human lung epithelial cells. Malignant human lung epithelial cells of lung carcinoma origin (A459, ATCC^®^ CCL-185TM, ATCC, Manassas, VA, USA) and non-malignant human lung epithelial cells of normal lung/bronchus origin (BEAS-2B, ATCC^®^ CRL-9609TM, ATCC, Manassas, VA, USA) were used. Both cell types were cultured in Dulbecco’s modified Eagle’s medium (DMEM, PAN-Biotech GmbH, Aidenbach, Germany) supplemented with 10% fetal bovine serum (FBS, South America origin, PAN-Biotech GmbH, Aidenbach, Germany) and 1% penicillin/streptomycin (PAN-Biotech GmbH, Aidenbach, Germany). In addition, the culture medium for the BEAS-2B cells was supplemented with 1% glutamine (Gibco^®^ GlutaMAX^TM^, Life Technologies, Carlsbad, CA, USA). The cells were cultured in a humidified atmosphere at 37 °C with 5% CO_2_.

Unless stated otherwise, the cells were seeded in 96-well plates at 2000 cells/well in 200 µL of complete cell culture medium and cultured for 24 h before the treatment, i.e., the addition of the nanoparticles. All experiments were performed simultaneously on A549 and BEAS-2B cells. The nanoparticles were sterilized using UV light (~260 nm, 30 min) and added to the cells in the cell culture medium at a final concentration of 10 µg/mL. The incubation was followed for up to 4 days (depending on assay type). All experiments were run in triplicate.

#### 2.4.1. Cellular Uptake and Intracellular Release of Iron

Nanoparticle uptake by A549 and BEAS-2B cells was evaluated qualitatively using Prussian blue and eosin staining, and quantitatively by atomic absorption spectroscopy, electrothermal technique (AAS-ET).

##### Prussian Blue and Eosin Staining

A549 and BEAS-2B cells were seeded and treated with SPIONs or SPION@mSiO_2_, according to the standard procedure. After 24 h of incubation with the nanoparticles, the cells were washed two times with 200 µL of PBS to remove any non-absorbed nanoparticles, fixed for 20 min with 3.8% paraformaldehyde and washed again with 200 µL of PBS. The nanoparticles were stained using freshly prepared Prussian blue reagent (the aqueous solution of 5% potassium ferrocyanide in 10% hydrochloric acid) for 20 min [34]. The cells were washed three times with 200 µL of PBS. The cytoplasm was counterstained with 1% eosin (Sigma-Aldrich, St. Louis, MO, USA) for 5 min and the cells were once again rinsed with 200 µL of PBS. The bright field images of the cells were taken using an optical microscope (Axiovert 40 CFL, Zeiss, Oberkochen, Germany).

##### AAS-ET Analysis

A549 and BEAS-2B cells were seeded in T25 flat bottom tissue culture flasks at 150,000 cells/flask and allowed to attach to the bottom of the flask. SPIONs or SPION@mSiO_2_ were added to the cells 24 h after seeding at 20 µg/mL (the same ratio between the amount of the nanoparticles and the number of cells seeded). Twenty-four hours after the treatment, the cells were washed three times with 3 mL PBS, collected using 0.25% trypsin in 1 mM EDTA solution (PAN Biotech GmbH, Aidenbach, Germany) and counted using a Bürker chamber. The concentration of iron in cell suspensions was determined using AAS-ET. The differences in cell number were taken into consideration and thus total the iron content determined via AAS-ET was divided by the average number of cells for each sample. The experiment was performed in triplicate.

#### 2.4.2. Proliferation and Cytotoxicity

##### Real-Time Impedance Measurements

Cell proliferation was evaluated using a real-time impedance measurement method (xCelligence RTCA SP, ACEA Biosciences, Inc., San Diego, CA, USA). The cells were seeded at 2000 cells/well in 96-well plates with microelectronic cell sensor arrays integrated into the bottom of plates (E-Plate 96, ACEA Biosciences, Inc., San Diego, CA, USA). SPIONs and SPION@mSiO_2_ (10 µg/mL) were added to the cell culture medium 28 h after cell seeding. The incubation of the cells with the nanoparticles was continued for 3 days. Through the whole experiment, the plate was kept inside xCelligence RTCA SP apparatus placed in the incubator (5% CO_2_, 37 °C, humidified atmosphere) and the impedance was measured every 15 min. The registered impedance values were recalculated and presented as a normalized cell index using the provided xCelligence RTCA SP software (version 1.2.1).

##### Live/Dead Staining

Live/dead staining was performed after 3 days of incubation of the cells with SPIONs or SPION@mSiO_2_. The culture medium was replaced with FluoroBrite^TM^ DMEM (Gibco, Life Technologies, Carlsbad, CA, USA) containing 0.1% calcein AM (Sigma-Aldrich, St. Louis, MO, USA) and 0.1% propidium iodide (Sigma-Aldrich, St. Louis, MO, USA). The cells were incubated for 20 min. Fluorescent microscopy images were taken using a fluorescence microscope (Axiovert 40 CFL with HXP 120 C Metal Halide Illuminator, Zeiss, Oberkochen, Germany).

##### Lactate Dehydrogenase (LDH) Assay

The cytotoxicity of SPIONs and SPION@mSiO_2_ was evaluated using an LDH Assay Kit-WST (Dojindo, Kumamoto, Japan). Three days after treatment, 50 µL of cell culture supernatant was transferred to an optically clear 96-well plate and mixed with 100 µL of the working solution (prepared according to the manufacturer’s instructions briefly before the experiment). The plate was incubated in the dark for 30 min at room temperature. Then 50 µL of the stop solution was added into each well. The absorbance at 490 nm was measured using a microplate reader (VICTOR Multilabel Plate Reader, PerkinElmer, Waltham, MA, USA).

#### 2.4.3. Cell Migration

The cells were seeded and treated with the nanoparticles according to the standard procedure. During the whole experiment, the plates were kept inside an IncuCyte ZOOM System (Essen BioScience, Newark Close, UK) and phase contrast images were recorded every 2 h (four images for each well). The images were then analyzed and compiled into image sets showing the proliferation and migration of the cells in selected areas of the well plate (1280 µm × 1726 µm) using the provided IncuCyte software (version 2016A).

The prepared image sets were analyzed using ImageJ software [35]. The paths travelled by the cells were determined using the Manual Cell Tracking plug-in. The middle of the nuclei of the analyzed cell was marked and its XY coordinates were defined. The cell was followed through all subsequent frames. The difference in XY values between the frames allowed for the calculation of the distance travelled by the cell within two subsequent images (in µm). The total distance travelled by the cell was determined as a sum of all partitive distances. The velocity of the cell (in µm/h) was calculated as the distance between two subsequent frames divided by the time span between those images (2 h interval). For each sample (control, SPIONs or SPION@mSiO_2_ treated A549 or BEAS-2B cells) at least 20 cells originating from four different image sets were analyzed. Additionally, all of the cells visible in the images were counted using the Cell Counter plug-in (at least five image sets for each sample group).

### 2.5. Statistics

The statistical analyses of the obtained data were done using a one-way analysis of variance (one-way ANOVA) followed by Tukey’s post hoc test. The assumptions of normal distribution and equal variance were verified using the Shapiro–Wilk and Levene median tests, respectively (*p*-value < 0.05). The analyses were performed using SigmaPlot 12.3 software (Systat Software, Inc., San Jose, CA, USA). The results are presented as mean ± standard deviation (SD), unless stated otherwise.

## 3. Results

### 3.1. Nanoparticle Characterization

**Figure 2:**
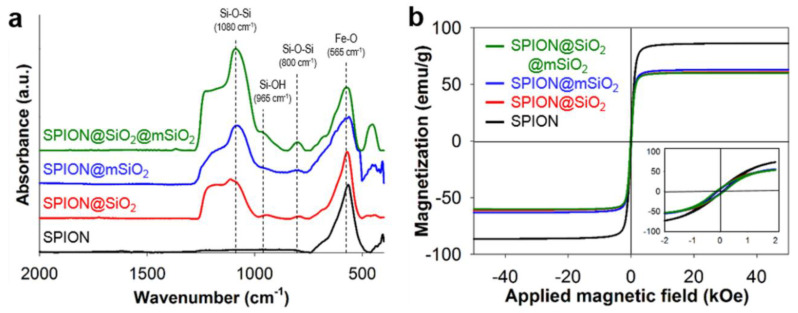
Fourier transform infrared spectroscopy (FTIR) spectra of the nanoparticles (**a**) and magnetization curves of the nanoparticles at 300 K (**b**).

Silica coatings were successfully deposited on the SPIONs as evidenced by FTIR (Figure 2a). The bands originating from the Fe-O bond (565 cm^−1^) were present in all spectra. The spectra of modified SPIONs exhibited the bands characteristic for SiO_2_ (Si-O-Si: 800 and 1080 cm^−1^; Si-OH: 965 cm^−1^). The intensity ratios between the major bands representing Fe-O and Si-O-Si bonds increased from 1:0.58 for SPION@SiO_2_ to 1:0.88 and 1:1.88 for SPION@mSiO_2_ and SPION@SiO_2_@mSiO_2_, respectively. This indicated the increasing amount of silica deposited on the SPIONs and the effective formation of multiple silica coatings on the SPIONs.

The characterization of the magnetic properties of the modified nanoparticles (Figure 2b and Table 1) showed that the highest magnetization saturation (M_S_) value was measured for the unmodified SPIONs (86.3 emu/g). The M_S_ decreased with the increase in SiO_2_ content in the nanoparticles (63.1 emu/g for SPION@SiO_2_, 61.0 emu/g for SPION@mSiO_2_ and 60.1 emu/g for SPION@SiO_2_@mSiO_2_). The differences in the M_S_ of the nanoparticles allowed for the determination of the SiO_2_ content. As expected, the highest SiO_2_ content was measured for SPION@SiO_2_@mSiO_2_ (30.4%), however the amount of SiO_2_ in both SPION@SiO_2_ and SPION@mSiO_2_ was above 25% (26.8% and 29.3%, respectively). The increase in SiO_2_ content resulted in the increase in the ratio between remanence and magnetization saturation (M_R_/M_S_) and coercivity (H_C_).

**Figure 3:**
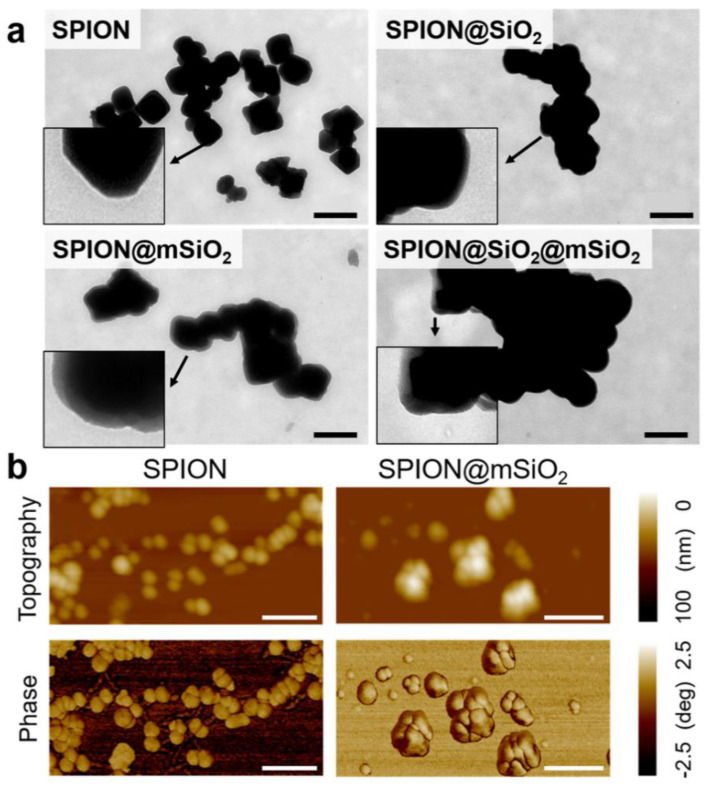
Transmission electron microscopy (TEM) images of the nanoparticles (scale bar = 300 nm, inserts show SPIONs at higher magnification) (**a**) and atomic force microscopy (AFM) topographical and phase images of SPIONs and SPION@mSiO_2_ (scale bar = 200 nm) (**b**).

TEM images of the nanoparticles (Figure 3a) showed that unmodified SPIONs were irregular in shape, with the diameters ranging from 50 to 200 nm. Silica layers were visible on the surface of all the modified SPIONs, however, the most uniform coating was obtained for SPION@mSiO_2_. AFM topography and phase images were also acquired (Figure 3b). According to the literature, drastic changes in phase angles could be correlated with sudden changes in surface stiffness and with the change in surface Young’s modulus [36]. In our study, no noticeable changes in AFM phase images were observed for SPION@mSiO_2_. This indicated that the deposited coatings were homogenous and uniform in terms of mechanical properties.

**Figure 4:**
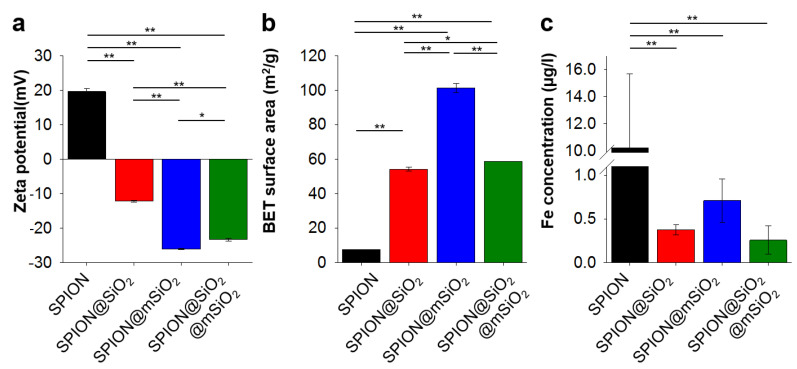
Zeta potential (**a**), Brunauer–Emmett–Teller (BET) surface area (**b**) and concentration of iron released from 1 mg of the nanoparticles (**c**). Statistically significant differences at * *p* < 0.05 and ** *p* < 0.001.

The modification of SPIONs with silica layers drastically changed the surface properties of the nanoparticles. The surface zeta potential (Figure 4a) was +19.6 ± 0.8 mV for unmodified SPIONs and it decreased to −12.2 ± 0.3 mV for SPION@SiO_2_ and −26.1 ± 0.1 mV for SPION@mSiO_2_. The BET surface area of the nanoparticles (Figure 4b) increased from 7.54 ± 0.02 m^2^/g for SPIONs to even 101.3 ± 2.8 m^2^/g for SPION@mSiO_2_. The coating of SPIONs with a silica layer decreased iron dissolution and its release in UHQ-water at least 10-fold (Figure 4c). A statistically significant difference in the concentration of iron released was observed between unmodified SPIONs and all silica coated ones.

### 3.2. In Vitro Cytotoxicity

**Figure 5:**
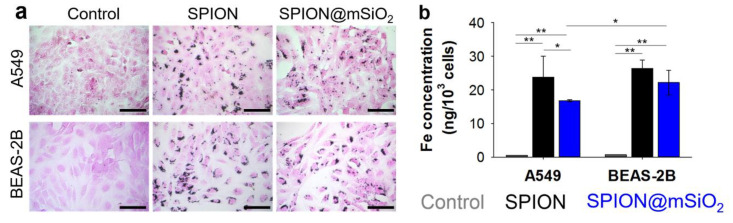
Prussian blue and eosin staining of A549 and BEAS-2B cells incubated with SPIONs or SPION@mSiO2 for 24 h. Scale bar = 100 μm (**a**) and concentration of Fe in the cells (**b**). Statistically significant differences at * *p* < 0.05 and ** *p* < 0.001.

SPIONs and SPION@mSiO_2_ were tested in contact with human lung epithelial cells of carcinoma (A549) and normal (BEAS-2B) origin. Both types of the nanoparticles were effectively incorporated by A549 and BEAS-2B cells. The agglomerates of incorporated nanoparticles were clearly visible in optical microscopy images (Figure 5a). As visualized by Prussian blue and eosin staining, the majority of the nanoparticles were stored in the areas in close proximity to cell nuclei. Regardless of the presence of nanoparticles, the cells exhibited typical morphology. The concentration of iron determined by AAS-ET (Figure 5b) in the control cells was 0.53 ± 0.02 ng/10^3^ cells for A549 cells and 0.67 ± 0.02 ng/10^3^ cells for BEAS-2B. A significant increase in iron concentration was observed for both A549 and BEAS-2B cells treated with SPIONs or SPION@mSiO_2_ (at least a 30-fold increase). In both cell types, the content of iron was higher in the case of the SPIONs due to the fact that the nanoparticles were delivered to the cells in an equal mass concentration, while almost 30% of the weight of SPION@mSiO_2_ was accrued by SiO_2_. BEAS-2B cells incorporated a significantly higher amount of SPION@mSiO_2_ in comparison with A549 cells. No statistically significant differences were observed between BEAS-2B and A549 cells treated with unmodified SPIONs.

**Figure 6:**
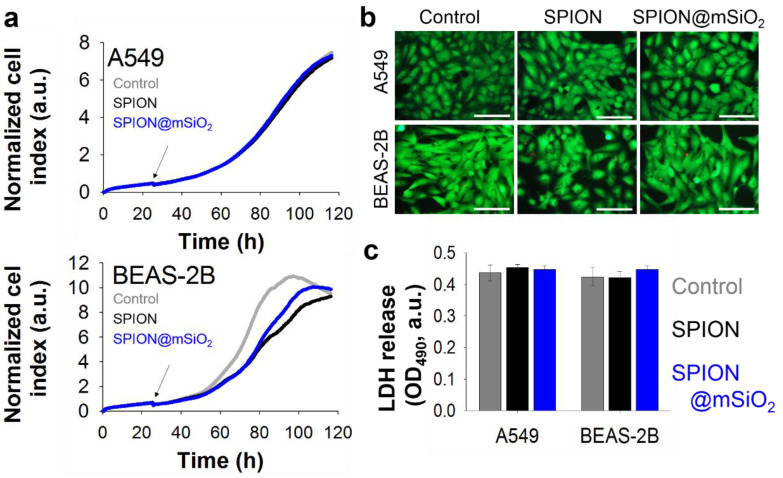
Proliferation profiles (arrows indicate addition of nanoparticles) (**a**), live/dead images (scale bar = 100 μm) (**b**) and lactate dehydrogenase (LDH) release (**c**) of A549 and BEAS-2B cells incubated with 10 µg/mL SPIONs or SPION@mSiO_2_ for 3 days.

Proliferation profiles of the cells incubated with the suspension of nanoparticles (Figure 6a) showed that the growth of A549 cells was not obstructed by the presence of either SPIONs or SPION@mSiO_2_. Surprisingly, the proliferation of BEAS-2B cells was hampered after the addition of the nanoparticles. Untreated (control) BEAS-2B cells reached the maximal values of the cell index (indicating full confluence) after 86–90 h. Nanoparticle-treated BEAS-2B cells did not fully cover the available surface until the end of experiment, although their proliferation increased in the last 20 h of incubation. Live/dead staining performed after 3 days of incubation with nanoparticles (Figure 6b) did not show an increased number of dead cells in the case of BEAS-2B cells. All cells exhibited typical morphology that did not differ from control cells. The levels of released LDH (Figure 6c) were similar for all samples; no statistically significant differences were observed.

**Figure 7:**
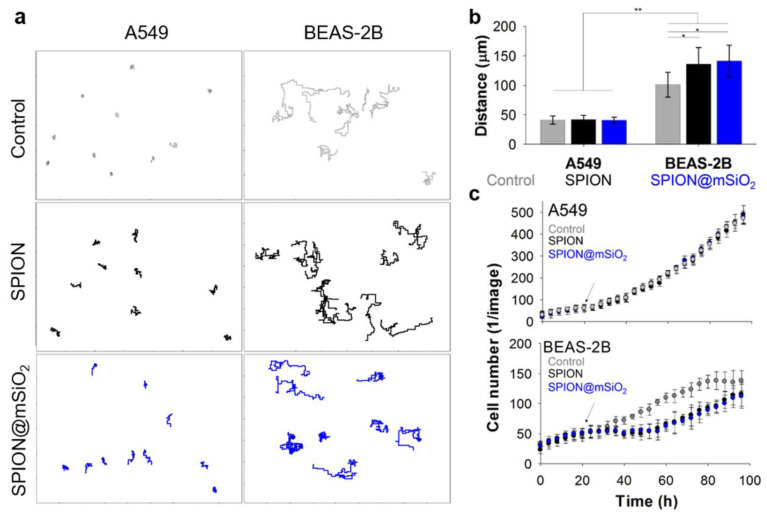
Line traces (after the treatment) (**a**), total distance travelled (**b**) and cell number (**c**) of A549 and BEAS-2B cells incubated with SPIONs or SPION@mSiO_2_. Statistically significant differences at * *p* < 0.05 and ** *p* < 0.001.

The analysis of cell migration revealed significant differences between A549 and BEAS-2B cells (Videos—Supplementary Materials). BEAS-2B cells migrated more extensively than A549 cells (Figure 7a).

The average total distance travelled (Figure 7b) by untreated A549 cells was 41 ± 7 µm and for control BEAS-2B cells it was 100 ± 21 µm. The addition of SPIONs or SPION@mSiO_2_ did not influence the migration of A549 cells (total distances were 42 ± 7 µm and 41 ± 5 µm, respectively). In the case of BEAS-2B cells treated with SPIONs and SPION@mSiO_2_, the length of the cell paths increased to 135 ± 28 µm and 140 ± 27 µm, respectively. The average velocity of nanoparticle-treated A549 cells was comparable to untreated cells, regardless of nanoparticle type (between 0.55–0.59 µm/h). The addition of either SPIONs or SPION@mSiO_2_ significantly increased the average velocity of BEAS-2B cells (control cells: 1.31 ± 0.26 µm/h, SPIONs: 1.75 ± 0.36 µm/h, SPION@mSiO_2_: 1.81 ± 0.35 µm/h).

The same phase contrast images were used for the analysis of cell number (Figure 7c). SPION@mSiO_2_-treated A549 cells were proliferating at the same rate as their untreated counterparts. The number of A549 cells was not influenced by the addition of nanoparticles. On the contrary, significant differences were found for BEAS-2B cells. Before the treatment, the growth of BEAS-2B cells in both groups was at the same level. After the addition of the nanoparticles, the number of BEAS-2B cells was constant for the initial 24–28 h post treatment. Then, the proliferation of BEAS-2B cells increased again and neared to the level of untreated cells by the end of the experiment. Untreated BEAS-2B cells proliferated at steady rate up to 84 h (almost 100% confluence). During the first 24 h after the treatment, the migration of BEAS-2B cells intensified and the cell number did not change until all SPION@mSiO_2_ agglomerates were incorporated by the cells.

## 4. Discussion

The following study was aimed at the development of versatile SPIONs modified with silica coatings for the prevention of iron release and the creation of a platform for the diagnosis and therapy of lung cancer. Silica coatings were successfully deposited on the SPIONs. The chemical composition of all modified SPIONs was similar, as proved by FTIR. The amount of silica deposited on the SPIONs varied slightly between the samples and it was the highest for SPION@SiO_2_@mSiO_2_, as evidenced by both FTIR and the analysis of magnetic properties. The most uniform coating was obtained for SPION@mSiO_2_, as evidenced by TEM analysis.

The presence of a silica shell decreased the magnetization of the modified SPIONs. The saturation magnetization decreased in inverse proportion to the amount of silica deposited on the SPIONs. The changes in saturation magnetization of the modified SPIONs were similar to those reported by others [28,37,38] and the magnetic properties of all the modified SPIONs were comparable with other materials used in hyperthermia: minimal M_S_, adaptable for biomedical purposes, were reported to be 7–22 emu/g [13,39]. The thickness of the silica layer can be modified by adjusting the amount of TEOS used for the sol–gel process, as evidenced by Deng et al. [28]. A higher amount of TEOS, i.e., a silica precursor, resulted in the formation of thicker coatings. However, the same study reported that the magnetic properties of silica-coated SPIONs deteriorated proportionally to the increased coating thickness.

Deposited coatings significantly influenced the surface properties of the SPIONs. The most pronounced changes were observed for SPION@mSiO_2_. A single @mSiO_2_ coating effectively inhibited the dissolution of iron and its release from the nanoparticles (more than 10-fold decrease compared to the unmodified SPIONs). The prevention of iron release from the SPIONs was of utmost importance, since iron is considered as a cofactor in cancer cell proliferation [23,40]. SPION@mSiO_2_ nanoparticles were characterized by the highest surface area and the lowest surface zeta potential among all the modified SPIONs. Interestingly, the highest surface area was observed for SPION@mSiO_2_, while the surface area of SPION@SiO_2_ and SPION@SiO_2_@mSiO_2_ were at a similar level. Considering that the BET surface area was dependent on the outermost layer, it was expected that the surface area of SPION@SiO_2_@mSiO_2_ should have been close to SPION@mSiO_2_. However, the results of the magnetic properties testing showed that the deposition of an mSiO_2_ coating on SPION@SiO_2_ increased the total silica content in SPION@SiO_2_@mSiO_2_ by only approximately 3.6%, as compared to SPION@SiO_2_. This indicated that the deposition of mSiO_2_ on SPION@SiO_2_ was less successful than on the unmodified SPIONs. The percentage share of mSiO_2_ in SPION@SiO_2_@mSiO_2_ was significantly lower than in SPION@mSiO_2_, thus the increase in the BET surface area was less prominent. The coating was uniform in terms of its mechanical properties and integrity, as evidenced by AFM topography and phase imaging.

SPION@mSiO_2_ offered the highest surface area available for prospective loading with biologically active molecules (e.g., peptides, siRNA) and the sufficient inhibition of iron release (no statistically significant differences in iron release between SPION@SiO_2_, SPION@mSiO_2_ and SPION@SiO_2_@mSiO_2_). In addition, the formation of an mSiO_2_ layer on SPION@SiO_2_ was less successful, as evidenced by the measurements of the magnetic properties. The deposition of an mSiO_2_ layer on SPION@SiO_2_ nanoparticles, leading to the formation of SPION@SiO_2_@mSiO_2_, increased the silica content only by 3.6% (to compare—the percentage silica share in SPION@mSiO_2_ was 29.3%). Thus, SPION@SiO_2_ was selected from all the modified nanoparticles for further in vitro studies aimed at the evaluation of its cytotoxicity in contact with human lung epithelial cells.

The uptake of SPIONs and SPION@mSiO_2_ by human lung epithelial cells of carcinoma (A549) and normal (BEAS-2B) origin was evaluated at the beginning. As shown by Prussian blue and eosin staining as well as AAS-ET measurements, both types of the nanoparticles were efficiently incorporated by the cells. The nanoparticles agglomerated inside the cells and the majority of them were accumulated around the nuclei. Although the detailed mechanism of the nanoparticle uptake was not the objective of the following research, the information on the ways in which the nanoparticles enter the cells can be found in the literature. Kim et al. [10] analyzed the uptake by A549 cells of cobalt ferrite magnetic nanoparticles covered with a silica layer. The uptake of the nanoparticles was blocked at a low temperature (4 °C) or in the presence of certain metabolic inhibitors (including sodium azide, sucrose and bafilomycin A). It was demonstrated that the nanoparticles were internalized by the cells through an energy-dependent endosomal–lysosomal mechanism.

The presence of the nanoparticles strongly influenced the proliferation of BEAS-2B cells, while it did not affect A549 cells. It is noteworthy that no signs of increased cytotoxicity or irritation were observed, indicating that neither the SPIONs nor SPION@mSiO_2_ damaged the cells. The levels of LDH released to the cell culture medium in nanoparticle-treated BEAS-2B cells were the same as for the control cells.

The noticeable differences were found in the behavior of A549 and BEAS-2B cells incubated with the nanoparticles. The analyses were focused only on the time after the addition of the nanoparticles. In general, the motility of the A549 cells was significantly lower than that of BEAS-2B cells. One of the reasons for that could be the different morphology of these two cell types. A549 cells are small, carcinoma-derived cells growing closely with each other and forming colonies characteristic of malignant cells. BEAS-2B cells are of normal origin, they are much bigger than A549 cells, they have widespread cytoplasm and they do not tend to form clusters. Around the clock analysis of cell behavior showed that the A549 cells were growing normally and incorporated the nanoparticles once they were in close proximity to the cell. On the other hand, the BEAS-2B cells migrated more extensively, covering greater areas of the well, being able to incorporate almost all of the nanoparticles within the first 24 h post treatment. The results of the cell migration analyses confirmed that the decrease in proliferation of the BEAS-2B cells in the presence of SPIONs or SPION@mSiO_2_ was not caused by the cytotoxicity of the nanoparticles. To fully understand this phenomenon, additional studies focusing on the influence of the nanoparticles on e.g., the cell cycle and the expression of pro-inflammatory cytokines (i.e., IL-6) and actin-bundling protein (i.e., fascin-1), are necessary [41]. The increased migration of the BEAS-2B cells may be associated with their natural tendency for remodeling. In response to an injury, the airway epithelium can undergo a remodeling process [42]. It may be related to the repair of any structural changes that can occur during lung development or after an acute injury. It is a natural and physiological process that allows for the restoration of the normal airway structure and function. However, chronic lung injuries or inflammation (e.g., in asthma patients) can lead to pathological airway remodeling, leading to wall thickening, the hypertrophy of smooth muscle cells and the hypersecretion of mucus [43].

The developed silica-coated SPIONs can be also considered as a versatile carrier used in lung cancer treatment, either alone or as a component of composite inhalable drug delivery systems. The latter is particularly interesting, as the addition of magnetic nanoparticles in polymeric [44] or lipid [45,46] drug delivery carriers or liposomes [47] allows for guided accumulation in the desired area of the lungs with the use of an external magnetic field. Our team had already used SPION@mSiO_2_ as a component of the inhalable fatty acid-based microparticles loaded with paclitaxel [46]. The reasoning behind such a design for the drug carrier system was as follows: upon inhalation, the microparticles will be drawn to the tumor site using an external magnetic field. Then an alternating electromagnetic field will be applied, which will lead to the heating up of the magnetic nanoparticles, the melting of the fatty acid matrix of the microparticles and in consequence the release of paclitaxel directly to the tumor site. The composite microparticles had physicochemical properties suitable for such an application (e.g., appropriate size, melting temperature and mobility in a magnetic field). In vitro studies showed the efficient inhibition of A549 malignant lung epithelial cells, while empty microparticles or those loaded only with SPION@mSiO_2_ did not influence the cell viability, paving the way for the potential application of a developed system in lung cancer treatment.

## 5. Conclusions

In this study, SPIONs were modified with silica coatings of different morphologies and the physicochemical properties of the obtained nanoparticles were characterized by various techniques. The magnetic properties of modified SPIONs changed but remained adequate for hyperthermia treatment. It was shown that the presence of a silica coating significantly inhibited the release of iron from SPIONs and thus enabled the use of such nanoparticles for the diagnosis and treatment of cancer. At the same time, the applied coatings increased the surface area of SPIONs, providing a platform for further loading of drugs or other moieties. In the end, SPIONs and SPIONs coated with a single mesporous silica layer were proven to be cytocompatible with lung epithelial cells. The developed SPION-based carriers can be enriched in anticancer drugs or biologically active molecules and administered to the patient e.g., via inhalation directly to the lung tumor. It is expected that magnetic hyperthermia treatment coupled with the delivery of cancer suppression factors will increase the efficacy of the treatment.

## Figures and Tables

**Table 1:** Magnetic properties of the nanoparticles (M_S_—magnetization saturation, M_R_—remanence, H_C_—coercivity).

Sample	M_s_ (emu/g)	Silica Content at 300K (wt %)	M_R_/M_S_	H_C_ (Oe)
SPIONs	86.3	-	0.063	92
SPION@SiO_2_	63.1	26.8	0.084	104
SPION@mSiO_2_	61.0	29.3	0.109	123
SPION@SiO_2_@mSiO_2_	60.1	30.4	0.120	143

## Supplementary Materials

The following are available online at https://www.mdpi.com/2079-4991/10/6/1076/s1, Video S1: Growth of A549 control cells, Video S2: Growth of A549 cells incubated with 10 µg/mL of SPION, Video S3: Growth of A549 cells incubated with 10 µg/mL of SPION@mSiO_2_, Video S4: Growth of BEAS-2B control cells, Video S5: Growth of BEAS-2B cells incubated with 10 µg/mL of SPION, Video S6: Growth of BEAS-2B cells incubated with 10 µg/mL of SPION@mSiO_2_.

## References

[1] Laurent S., Forge D., Port M., Roch A., Robic C., Elst L.V., Muller R.N. (2008). Magnetic iron oxide nanoparticles: Synthesis, stabilization, vectorization, physicochemical characterizations, and biological applications. Chem. Rev..

[2] Gupta A.K., Gupta M. (2005). Synthesis and surface engineering of iron oxide nanoparticles for biomedical applications. Biomaterials.

[3] Gobbo O.L., Wetterling F., Vaes P., Teughels S., Markos F., Edge D., Shortt C.M., Crosbie-Staunton K., Radomski M.W., Volkov Y. (2015). Biodistribution and pharmacokinetic studies of SPION using particle electron paramagnetic resonance, MRI and ICP-MS. Nanomedicine.

[4] Yan L., Luo L., Amirshaghaghi A., Miller J., Meng C., You T., Busch T.M., Tsourkas A., Cheng Z. (2019). Dextran-benzoporphyrin derivative (BPD) coated superparamagnetic iron oxide nanoparticle (SPION) micelles for T2-weighted magnetic resonance imaging and photodynamic therapy. Bioconjugate Chem..

[5] Pöttler M., Schreiber E., Dürr S., Döllinger M., Alexiou C. (2018). Regenerative medicine of the vocal fold: Magnetic tissue engineering (MTE) using superparamagnetic iron oxide nanoparticles. Laryngo Rhino Otol..

[6] Li C., Armstrong J.P., Pence I.J., Kit-Anan W., Puetzer J.L., Carreira S.C., Moore A.C., Stevens M.M. (2018). Glycosylated superparamagnetic nanoparticle gradients for osteochondral tissue engineering. Biomaterials.

[7] Adams S.A., Hauser J.L., Allen A.L.C., Lindquist K.P., Ramirez A.P., Oliver S., Zhang J.Z. (2018). Fe_3_O_4_@ SiO_2_ nanoparticles functionalized with gold and poly (vinylpyrrolidone) for bio-separation and sensing applications. ACS Appl. Nano Mater..

[8] Tewes F., Ehrhardt C., Healy A.M. (2014). Superparamagnetic iron oxide nanoparticles (SPIONs)-loaded Trojan microparticles for targeted aerosol delivery to the lung. Eur. J. Pharm. Biopharm..

[9] Jeon H., Kim J., Lee Y.M., Kim J., Choi H.W., Lee J., Park H., Kang Y., Kim I.-S., Lee B.-H. (2016). Poly-paclitaxel/cyclodextrin-SPION nano-assembly for magnetically guided drug delivery system. J. Control. Release.

[10] Amirshaghaghi A., Yan L., Miller J., Daniel Y., Stein J.M., Busch T.M., Cheng Z., Tsourkas A. (2019). Chlorin e6-coated superparamagnetic iron oxide nanoparticle (SPION) nanoclusters as a theranostic agent for dual-mode imaging and photodynamic therapy. Sci. Rep..

[11] Sun X., Liu B., Chen X., Lin H., Peng Y., Li Y., Zheng H., Xu Y., Ou X., Yan S. (2019). Aptamer-assisted superparamagnetic iron oxide nanoparticles as multifunctional drug delivery platform for chemo-photodynamic combination therapy. J. Mater. Sci. Mater. Med..

[12] Hayashi K., Nakamura M., Sakamoto W., Yogo T., Miki H., Ozaki S., Abe M., Matsumoto T., Ishimura K. (2013). Superparamagnetic nanoparticle clusters for cancer theranostics combining magnetic resonance imaging and hyperthermia treatment. Theranostics.

[13] Hergt R., Dutz S., Müller R., Zeisberger M. (2006). Magnetic particle hyperthermia: Nanoparticle magnetism and materials development for cancer therapy. J. Phys. Condens. Matter.

[14] Guardia P., Di Corato R., Lartigue L., Wilhelm C., Espinosa A., Garcia-Hernandez M., Gazeau F., Manna L., Pellegrino T. (2012). Water-soluble iron oxide nanocubes with high values of specific absorption rate for cancer cell hyperthermia treatment. ACS Nano.

[15] Ahmed K., Tabuchi Y., Kondo T. (2015). Hyperthermia: An effective strategy to induce apoptosis in cancer cells. Apoptosis.

[16] Gupta A.K., Naregalkar R.R., Vaidya V.D., Gupta M. (2007). Recent advances on surface engineering of magnetic iron oxide nanoparticles and their biomedical applications. Nanomedicine.

[17] Lachowicz D., Kaczyńska A., Wirecka R., Kmita A., Szczerba W., Bodzoń-Kułakowska A., Sikora M., Karewicz A., Zapotoczny S. (2018). A hybrid system for magnetic hyperthermia and drug delivery: SPION functionalized by curcumin conjugate. Materials.

[18] Mansouri M., Nazarpak M.H., Solouk A., Akbari S., Hasani-Sadrabadi M.M. (2017). Magnetic responsive of paclitaxel delivery system based on SPION and palmitoyl chitosan. J. Magn. Magn. Mater..

[19] Hauser A.K., Anderson K.W., Hilt J.Z. (2016). Peptide conjugated magnetic nanoparticles for magnetically mediated energy delivery to lung cancer cells. Nanomedicine.

[20] Abdelaziz H.M., Gaber M., Abd-Elwakil M.M., Mabrouk M.T., Elgohary M.M., Kamel N.M., Kabary D.M., Freag M.S., Samaha M.W., Mortada S.M. (2018). Inhalable particulate drug delivery systems for lung cancer therapy: Nanoparticles, microparticles, nanocomposites and nanoaggregates. J. Control. Release.

[21] Sadhukha T., Wiedmann T.S., Panyam J. (2013). Inhalable magnetic nanoparticles for targeted hyperthermia in lung cancer therapy. Biomaterials.

[22] Price D.N., Stromberg L.R., Kunda N.K., Muttil P. (2017). In vivo pulmonary delivery and magnetic-targeting of dry powder nano-in-microparticles. Mol. Pharm..

[23] Steegmann-Olmedillas J.L. (2011). The role of iron in tumour cell proliferation. Clin. Transl. Oncol..

[24] Anbarasu M., Anandan M., Chinnasamy E., Gopinath V., Balamurugan K. (2015). Synthesis and characterization of polyethylene glycol (PEG) coated Fe_3_O_4_ nanoparticles by chemical co-precipitation method for biomedical applications. Spectrochim. Acta Part A Mol. Biomol. Spectrosc..

[25] Strehl C., Maurizi L., Gaber T., Hoff P., Broschard T., Poole A.R., Hofmann H., Buttgereit F. (2016). Modification of the surface of superparamagnetic iron oxide nanoparticles to enable their safe application in humans. Int. J. Nanomed..

[26] Ma H.-l., Qi X.-r., Maitani Y., Nagai T. (2007). Preparation and characterization of superparamagnetic iron oxide nanoparticles stabilized by alginate. Int. J. Pharm..

[27] Keshtkar M., Shahbazi-Gahrouei D., Mehrgardi M., Aghaei M. (2018). Synthesis and cytotoxicity assessment of gold-coated magnetic iron oxide nanoparticles. J. Biomed. Phys. Eng..

[28] Deng Y.-H., Wang C.-C., Hu J.-H., Yang W.-L., Fu S.-K. (2005). Investigation of formation of silica-coated magnetite nanoparticles via sol–gel approach. Colloids Surf. A Physicochem. Eng. Asp..

[29] Figueira R., Silva C.J., Pereira E. (2015). Organic–inorganic hybrid sol–gel coatings for metal corrosion protection: A review of recent progress. J. Coat. Technol. Res..

[30] Hamzian N., Hashemi M., Ghorbani M., Bahreyni Toosi M.H., Ramezani M. (2017). Preparation, optimization and toxicity evaluation of (SPION-PLGA) ±PEG nanoparticles loaded with gemcitabine as a multifunctional nanoparticle for therapeutic and diagnostic applications. Iran. J. Pharm. Res..

[31] Mulens-Arias V., Rojas J.M., Sanz-Ortega L., Portilla Y., Pérez-Yagüe S., Barber D.F. (2019). Polyethylenimine-coated superparamagnetic iron oxide nanoparticles impair in vitro and in vivo angiogenesis. Nanomed. Nanotechnol. Biol. Med..

[32] Alpsoy L., Baykal A., Akal Z.Ü. (2018). Luteolin-loaded spion as a drug carrier for cancer cell in vitro. J. Supercond. Nov. Magn..

[33] Kim J., Kim H.S., Lee N., Kim T., Kim H., Yu T., Song I.C., Moon W.K., Hyeon T. (2008). Multifunctional uniform nanoparticles composed of a magnetite nanocrystal core and a mesoporous silica shell for magnetic resonance and fluorescence imaging and for drug delivery. Angew. Chem. Int. Ed..

[34] Zhu X.-M., Wang Y.-X.J., Leung K.C.-F., Lee S.-F., Zhao F., Wang D.-W., Lai J.M., Wan C., Cheng C.H., Ahuja A.T. (2012). Enhanced cellular uptake of aminosilane-coated superparamagnetic iron oxide nanoparticles in mammalian cell lines. Int. J. Nanomed..

[35] Schneider C.A., Rasband W.S., Eliceiri K.W. (2012). NIH Image to ImageJ: 25 years of image analysis. Nat. Methods.

[36] Magonov S., Elings V., Whangbo M.-H. (1997). Phase imaging and stiffness in tapping-mode atomic force microscopy. Surf. Sci..

[37] Deng Y., Qi D., Deng C., Zhang X., Zhao D. (2008). Superparamagnetic high-magnetization microspheres with an Fe_3_O_4_@SiO_2_ core and perpendicularly aligned mesoporous SiO_2_ shell for removal of microcystins. J. Am. Chem. Soc..

[38] Li K., Zeng Z., Xiong J., Yan L., Guo H., Liu S., Dai Y., Chen T. (2015). Fabrication of mesoporous Fe_3_O_4_@SiO_2_@CTAB–SiO_2_ magnetic microspheres with a core/shell structure and their efficient adsorption performance for the removal of trace PFOS from water. Colloids Surf. A Physicochem. Eng. Asp..

[39] Lee J.-H., Jang J.-T., Choi J.-S., Moon S.H., Noh S.-H., Kim J.-W., Kim J.-G., Kim I.-S., Park K.I., Cheon J. (2011). Exchange-coupled magnetic nanoparticles for efficient heat induction. Nat. Nanotechnol..

[40] Manz D.H., Blanchette N.L., Paul B.T., Torti F.M., Torti S.V. (2016). Iron and cancer: Recent insights. Ann. N. Y. Acad. Sci..

[41] Adams J.C. (2004). Roles of fascin in cell adhesion and motility. Curr. Opin. Cell Biol..

[42] Wang W.C., Kuo C.Y., Tzang B.S., Chen H.M., Kao S.H. (2012). IL-6 augmented motility of airway epithelial cell BEAS-2B via Akt/GSK-3β signaling pathway. J. Cell. Biochem..

[43] Fehrenbach H., Wagner C., Wegmann M. (2017). Airway remodeling in asthma: What really matters. Cell Tissue Res..

[44] Miranda M.S., Rodrigues M.T., Domingues R.M.A., Torrado E., Reis R.L., Pedrosa J., Gomes M.E. (2018). Exploring inhalable polymeric dry powders for anti-tuberculosis drug delivery. Mater. Sci. Eng. C.

[45] Upadhyay D., Scalia S., Vogel R., Wheate N., Salama R.O., Young P.M., Traini D., Chrzanowski W. (2012). Magnetised thermo responsive lipid vehicles for targeted and controlled lung drug delivery. Pharm. Res..

[46] Reczyńska K., Marchwica P., Khanal D., Borowik T., Langner M., Pamuła E., Chrzanowski W. (2020). Stimuli-sensitive fatty acid-based microparticles for the treatment of lung cancer. Mater. Sci. Eng. C.

[47] Nahar K., Absar S., Patel B., Ahsan F. (2014). Starch-coated magnetic liposomes as an inhalable carrier for accumulation of fasudil in the pulmonary vasculature. Int. J. Pharm..

